# Motor cortical stimulation for the treatment of trigeminal neuropathic pain secondary to an arteriovenous malformation. A case report.

**DOI:** 10.31053/1853.0605.v80.n3.41142

**Published:** 2023-09-29

**Authors:** Fernando Padilla-Lichtenberger, Florencia Belén Casto, Federico Garavaglia, Miguel Villaescusa, Carlos Ciraolo

**Affiliations:** 1 Servicio de Neurocirugía, Hospital Italiano de Buenos Aires Argentina; 2 Servicio de Neurocirugía, Sanatorio Allende Córdoba Argentina

**Keywords:** corteza motora, dolor facial, malformaciones arteriovenosas, neuralgia del trigémino, arteriovenous malformation, facial pain, motor cortex, trigeminal neuralgia, córtex motor, dor facial, malformações arteriovenosas, neuralgia do trigêmeo

## Abstract

**Introduction:**

Trigeminal neuropathic pain (TNP) is a syndrome of severe, disabling, constant facial pain arising from the trigeminal nerve or ganglion. Arteriovenous malformations (AVM) are a rare cause of TNP. The limited choices of intervention of TNP include peripheral nerve stimulation, trigeminal nucleotomy and motor cortex stimulation.

**Case report:**

We present a 56-year-old man who suffered from trigeminal neuropathic pain secondary to nerve compression due to a giant posterior fossa AVM. The pain was refractory to drug treatment. From all the therapeutic options available we declined the microvascular decompression of the trigeminal nerve due to the presence of the giant AVM, or stereotactic radiosurgery because of the AVM's diffuse nidus. After a multidisciplinary discussion we proposed a safe and reversible treatment: Motor Cortical Stimulation (MCS). We placed a 16-pole epidural electrode on the right precentral gyrus. The patient had satisfactory pain control with some supplemental medication. No complications or side effects such as seizures, sensory disturbances or infections were presented.

**Discussion:**

The limited choices of intervention of TNP include peripheral nerve stimulation, trigeminal nucleotomy and MCS. Henssen et al performed a systematic review where they investigated the effectiveness of MCS and discovered that this is significantly different among different chronic neuropathic orofacial pain disorders. A visual analogue scale (VAS) measured median pain relief of 66.5% was found.

**Conclusion:**

MCS should be one more tool to consider in highly selected cases, when other treatments are unfeasible.

## Resumen

**Introducción:**

El dolor neuropático trigeminal (DNT) es un síndrome de dolor facial intenso, incapacitante y constante que surge del nervio o ganglio del trigémino. Las malformaciones arteriovenosas (MAV) son una causa rara de DNT. Las opciones terapéuticas de DNT incluyen la estimulación de los nervios periféricos, la nucleotomía del trigémino y la estimulación cortical motora.

**Caso clínico:**

Presentamos el caso de un varón de 56 años con dolor neuropático trigeminal secundario a compresión nerviosa por una MAV gigante de fosa posterior. El dolor era refractario al tratamiento farmacológico. De todas las opciones terapéuticas disponibles, desestimamos la descompresión microvascular del nervio trigémino por la presencia de la MAV gigante, o la radiocirugía estereotáctica, por ser difuso el nido de la MAV. Tras una discusión multidisciplinar propusimos un tratamiento seguro y reversible: Estimulación cortical motora (ECM). Colocamos un electrodo epidural en el giro precentral derecho. El paciente tuvo un control satisfactorio del dolor con medicación suplementaria. No presentó complicaciones ni efectos secundarios como convulsiones, alteraciones sensoriales o infecciones.

**Discusión:**

Las opciones limitadas de intervención de DNT incluyen estimulación nerviosa periférica, nucleotomía trigeminal y ECM. Henssen et al realizaron una revisión sistemática donde investigaron la efectividad de MCS y descubrieron que esto es significativamente diferente entre los diferentes trastornos de dolor orofacial neuropático crónico. Se encontró un promedio de alivio del dolor medida por una escala analógica visual del 66,5%.

**Conclusión:**

La ECM debería ser una herramienta más a considerar en casos estrictamente seleccionados donde otros tratamientos no son viables.

## Resumo

**Introdução:**

A mudança climática global pode alterar a circulação do vírus sincicial respiratório A dor neuropática do trigêmeo (DNT) é uma síndrome de dor facial intensa, incapacitante e constante decorrente do nervo ou gânglio trigêmeo. As malformações arteriovenosas (MAV) são uma causa rara de PNT. As opções limitadas de intervenção do TNP incluem estimulação do nervo periférico, nucleotomia do trigêmeo e estimulação do córtex motor.

**Relato de caso:**

Apresentamos um homem de 56 anos que sofria de dor neuropática trigeminal secundária à compressão do nervo devido a uma MAV gigante da fossa posterior. A dor era refratária ao tratamento medicamentoso. De todas as opções terapêuticas disponíveis declinamos a descompressão microvascular do nervo trigêmeo pela presença da MAV gigante, ou a radiocirurgia estereotáxica pelo nidus difuso da MAV. Após discussão multidisciplinar propusemos um tratamento seguro e reversível: Estimulação Cortical Motora (ECM). Colocamos um eletrodo peridural de 16 polos no giro pré-central direito. A paciente apresentou controle satisfatório da dor com alguma medicação suplementar. Não foram apresentadas complicações ou efeitos colaterais como convulsões, distúrbios sensoriais ou infecções.

**Discussão:**

As opções limitadas de intervenção do TNP incluem estimulação do nervo periférico, nucleotomia trigeminal e MCS. Henssen et al realizaram uma revisão sistemática onde investigaram a eficácia do MCS e descobriram que isso é significativamente diferente entre diferentes distúrbios de dor orofacial neuropática crônica. Uma escala visual analógica mediu o alívio mediano da dor de 66,5%.

**Conclusão:**

A MCS deve ser mais uma ferramenta a ser considerada em casos altamente selecionados, quando outros tratamentos são inviáveis.

**CONCEPTOS CLAVE**

Qué se sabe sobre el tema.

Aunque no es frecuente, el dolor neuropático trigeminal puede ser causado por malformaciones arteriovenosas. Las opciones limitadas de intervención para el dolor neuropático trigeminal incluyen la estimulación de los nervios periféricos, la nucleotomía del trigémino y la estimulación de la corteza motora. Dada su rareza, el protocolo en casos refractarios a tratamientos convencionales sigue siendo controversial.

Qué aporta este trabajo.

Si bien está descrita la cirugía abierta, el tratamiento endovascular y la radiocirugía con gran éxito en el tratamiento de las MAV y el dolor neuropático consecuentemente, no está bien definido el manejo de los casos en los cuales estos tratamientos fallan. A nuestro conocimiento, no se ha descrito previamente un caso en el cual se haya empleado satisfactoriamente la estimulación cortical motora en pacientes con dolor neuropático trigeminal refractario secundario a MAV. Al día de hoy, no existe un consenso sobre el tratamiento óptimo para estos casos.

**Divulgación**

El dolor neuropático trigeminal es un tipo de dolor facial que puede ser muy invalidante. La malformación arteriovenosa es un nido anormal de vasos sanguíneos donde la sangre arterial fluye directamente hacia las venas de drenaje sin los capilares interpuestos normales. Constituyen una causa infrecuente de dolor neuropático trigeminal. Dada su rareza, el protocolo en casos que no responden a tratamientos convencionales sigue siendo controversial. Presentamos un caso de dolor trigeminal secundario a una malformación arteriovenosa, que no respondió a múltiples tratamientos, donde utilizamos la estimulación de la corteza motora cerebral con electrodos, obteniendo una gran mejoría del dolor y de la calidad de vida del paciente.

## Introduction

Trigeminal neuropathic pain (TNP) is a syndrome of stabbing, flaming, disabling facial pain arising from the trigeminal nerve or ganglion. Common etiologies include stroke, postherpetic neuralgia, iatrogenic injuries from sinus or dental surgery, facial trauma, intentional surgical lesioning (deafferentation), as well as local pathologic conditions (arterial or venous vascular conflict). Arteriovenous malformation (AVM) is an abnormal collection of blood vessels where arterial blood flows directly into draining veins without the normal interposed capillary beds. Around 10% of them are located at the posterior fossa. AVM are a rare cause of TNP, rates vary from 0.24 to 1.78% ^1^ . The pharmacologic treatment for TNP includes a variety of analgesics, as well as anticonvulsants (such as carbamazepine) and antidepressants. Adequate pain relief is hard to achieve with medical therapy. When this treatment fails, surgery should be considered ^2^ . In 1991, Tsubokawa et al. first reported electrical stimulation of the primary motor cortex for central pain syndromes. The indications for motor cortex stimulation (MCS) include poststroke pain, trigeminal neuropathic or deafferentation pain, brachial plexus avulsion, phantom limb pain, and various central and peripheral neuropathic pain syndromes ^3^ . In different series MCS outcomes have been variable, but application of the technique to TNP has yielded success rates of 75% to 100% ^3^ . We present a patient with TNP secondary to a giant cerebellar AVM that was treated with MCS.

## Case Report

**Figure 1:**
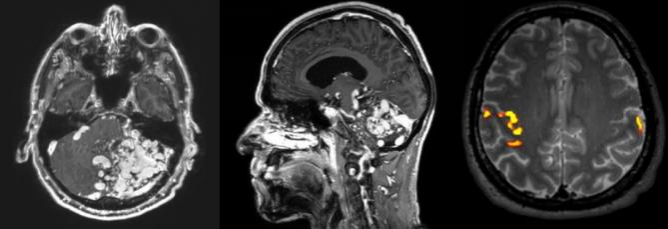
Cerebral MRI with gadolinium: A large AVM is centered in the left cerebellar hemisphere extending towards the middle cerebellar peduncle and left lateral margin of the pons. It has afferent branches of the basilar trunk, right vertebral artery and left posterior cerebral artery, and efferent venous branches projected towards the vein of Galen and left transverse sinus. It generates compression and displacement of the brainstem to the right and compression of the left trigeminal nerve and the left statoacoustic bundle. On the right, functional MRI showing right motor cortex.

We present a 56-year-old man who suffered from trigeminal neuropathic pain secondary to nerve compression due to a giant posterior fossa AVM. He consulted ten years ago for intermittent, paroxysmal, severe, electrical, and triggerable left V2-V3 pain episodes. Initially, he was examined in another center, where the diagnosis of a left cerebellar AVM Spetzler-Martin grade V was made (Figure 1). Throughout these ten years a total of five attempts of embolization were made. None of them achieved a complete occlusion of the AVM. Furthermore, the patient suffered left side facial palsy (House & Brackmann grade III), dysmetria, unsteady gait and progression of the TNP due to post-embolization sequelae.

When the patient consulted in our center, he was unable to control pain with 1200 mg of carbamazepine per day. He had already been under treatment with pregabalin 225 mg per day, acetaminophen 3000 mg per day, amitriptyline 50 mg per day and tramadol 200 mg per day. From all the therapeutic options available, we declined the microvascular decompression of the trigeminal nerve due to the presence of the giant AVM, or stereotactic radiosurgery because of the AVM's diffuse nidus. We analyzed that any procedure through the foramen ovale, such as radiofrequency or neuropraxia, was risky because of an anomalous vein drainage located near the Gasserian ganglion. After a multidisciplinary discussion, with the ethics committee approval, we proposed a safe and reversible treatment, although it required a larger approach and involved greater risk: MCS.

## Surgical Procedure

**Figure 2:**
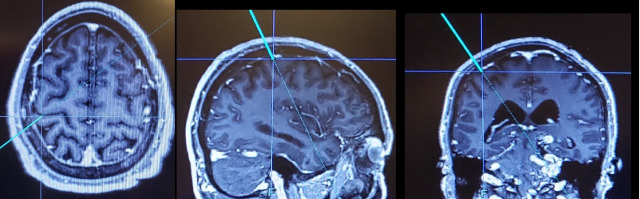
Neuronavigation system: (Stryker® Neuronavigation System, Stryker Corporation, Kalamazoo, U.S.A) showing the characteristic omega or epsilon shape on the surgical planning of the central sulcus.

**Figure 3:**
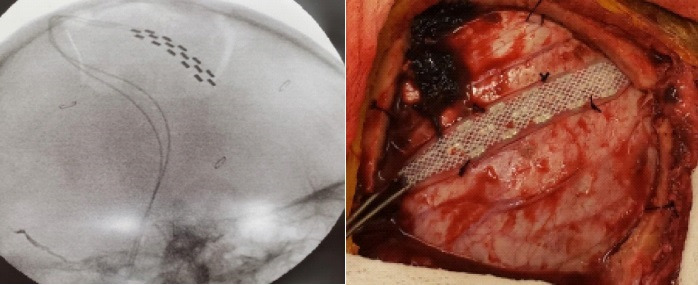
Intraoperative Imaging: Intraoperative fluoroscopy showing the correct placement of the electrode following the central sulcus. Surgical field showing the electrode at the central sulcus.

Before the procedure, an informed consent was signed. The patient underwent general anesthesia. We identified the central sulcus by using classical anatomic landmarks (4 cm behind the coronal fissure, contralateral to the painful area, 1 to 3 cm lateral to the midline; or the Kronlein's anatomical measurements: a line from the nasion to the inion, divided in 2 + 2 cm to a point at the middle of the zygomatic process) and by searching for its characteristic omega shape on the surgical planning station of our neuronavigation system (Stryker® Neuronavigation System, Stryker Corporation, Kalamazoo, U.S.A) (figure 2). We did a right horseshoe incision centered in the central sulcus and a craniotomy of 8 x 5 cm. Perioperatively, we confirmed the localization of the central sulcus with the neuronavigation system, and placed a 16-pole 565 Medtronic® Surgical Epidural Electrode (Medtronic®, Minneapolis, USA) on the right precentral gyrus (figure 3). We identified the correct location of the central fissure by recording somatosensory evoked potentials through the electrode in response to stimulation of the painful area. The N20/P20 wave inversion on somatosensory evoked potentials was obtained. Finally, the electrode was fixed at four sites on the dura mater at a site corresponding to the omega shape portion of the central sulcus (figure 2), to the motor cortex corresponding to the representation of the face. The main axis of the electrode was placed parallel to the direction of the central sulcus. Afterwards, we implanted the generator (Medtronic®, Minneapolis, USA) in the subcutaneous area of the right infraclavicular space in the same surgical act.

## Postoperative Course

A tonic stimulation with bipolar configuration was programmed. It consists of two programs that stimulate different areas.

Program 1: Voltage: 1.0mA. Pulse width: 120μs. Frequency: 80Hz

Program 2: Voltage: 0.4mA. Pulse width: 120μs. Frequency: 80Hz.

Patient was evaluated with a short form of Mc Gill pain questionnaire preoperatively and postoperatively at 3 and 6 months (Table 1). The patient had satisfactory pain control with 400 mg Carbamazepine per day. No complications or side effects such as seizures, sensory disturbances or infections were presented. The motor disability also improved (House & Brackmann Facial Palsy Grade II).

**Table 1:** Mc Gill pain questionnaire preoperatively and postoperatively at 3 and 6 months after the implantation. Satisfactory pain control after MCS.

	BEFORE SURGERY	3 MONTHS AFTER	6 MONTHS AFTER
*THROBBING*	0	1	1
*SHOOTING*	3	2	1
*STABBING*	3	2	0
*SHARP*	2	1	1
*CRAMPING*	0	1	1
*GNAWING*	1	0	0
*HOT BURNING*	0	0	1
*ACHING*	2	1	1
*HEAVY*	1	0	0
*TENDER*	3	1	1
*SPLITTING*	3	2	0
*TIRING*	2	1	1
*SICKENING*	1	0	0
*FEARFUL*	0	0	0
*PUNISHING*	2	1	0
*TOTAL*	23	13	9
			
*PRESENT PAIN INTENSITY (PPI)*			
*0= NO PAIN*			
*1= MILD*			
*2= DISCOMFORTING*			
*3= DISTRESSING*			
*4= HORRIBLE*			
*5= EXCRUTIATING*			
*VISUAL ANALOGUE SCALE (VAS)*			
90 60 40			

## Discussion

TNP is described in the International Classification of Orofacial Pain (ICOP), as a facial pain in the distribution of one or more branches of the trigeminal nerve caused by another disorder and indicative of neural damage. The primary pain is usually continuous or near-continuous, described as burning, squeezing, pins or needles. Although brief pain paroxysms may occur, these are not the predominant type. This classification distinguishes TNP from the subtypes of secondary trigeminal neuralgia. Even when secondary causes attributable to both trigeminal neuralgia and neuropathy are similar (multiple sclerosis, tumors, skull-base bone deformity, connective tissue diseases, arteriovenous malformation, dural arteriovenous fistula), clinical features and response to carbamazepine are distinct characteristics ^1^ . The available options for treatment of the medical refractory facial pain includes nondestructive approaches such as microvascular decompression (MVD), percutaneous interventions (radiofrequency ganglion lysis, glycerol injection, balloon compression), stereotactic radiosurgery and neuromodulation surgeries (peripheral and central neurostimulation) ^4^ . According to the International Headache Classification 3rd version (ICHD-3), our patient's symptoms fit into the category of "Painful trigeminal neuropathy", because the pain is continuous with detectable sensory deficits within the trigeminal distribution. Allodynic areas are much larger than the punctate trigger zones present in trigeminal neuralgia. The patient could carry out a normal active life. But after the diagnosis of the AVM, and promptly of the embolization, the pain became constant and significantly increased its intensity. There were no triggers as it was prior to embolization, and also added a left central facial palsy House & Brackmann grade III.

In our case, the microsurgery of this AVM was not recommended ^4^ . The limited choices of intervention of TNP include peripheral nerve stimulation, trigeminal nucleotomy and motor cortex stimulation (MCS). Transcranial direct current stimulation (tDCS) was an alternative. This stimulation is able to promote relief of pain, both short and long term (at least up to 7 days) after the end of the tDCS treatment ^5^ . Nevertheless, it was not the best option for our patient because tDCS works better in paroxysmal rather than continuous pain ^6^ . When pain becomes chronic, tDCS modulation might be less prominent. Hagenacker et al reported a relatively high drop out rate of tDCS, even though the tolerability of participating patients was high ^6^ . Peripheral nerve stimulation was discarded as well because our patient had a facial nerve palsy. Trigeminal nucleotomy was not chosen because it supposed an elevated risk in our particular case. This is the reason why we preferred doing MCS. However, this procedure does have its risks ^1^ . Rasche et al in a prospective study of 36 patients who underwent motor cortical stimulation, reported 4 patients with wound infection and 2 patients with technical failures (such as breakage of the cables of the leads or extensions), all of them required revision surgery (23%). Fontaine et al did a systematic review of the literature which included 157 patients. Hardware-related problems occurred in 5.1% of the patients, and infections in 5.7%. This usually required the removal of parts of or the entire MSC system. Seizures during the operation or the postoperative stimulation trial were reported in 12%, nevertheless no seizures or epilepsy have been reported after long-term follow-up. Two patients developed transient neurological deficits (1 speech disorder and 1 motor deficit). Two epidural and two subdural hematomas were also reported ^7^ . Even though these complications are described, the risks and complication rates of the procedure are justifiably low ^1^ .

Henssen et al introduced the concept of Chronic neuropathic orofacial pain (CNOP) ^8^ , a heterogeneous group of orofacial pain disorders, which can lead to different responses to MCS. They performed a systematic review where they investigated the effectiveness of MCS. They discovered that this is significantly different among different CNOP disorders. A visual analogue scale (VAS) measured median pain relief of 66.5%. Linear mixed-model analysis showed that patients suffering from trigeminal neuralgia responded significantly more favorably to MCS than patients suffering from dysfunctional pain syndromes. Also, patients suffering from CNOP caused by (supra)nuclear lesions responded marginally significantly better to MCS than patients suffering from CNOP due to trigeminal nerve lesions. García-Larrea and Peyron established that MCS appears to trigger rapid and phasic activation in the lateral thalamus, which leads to a cascade of prolonged events in medial thalamus, anterior cingulate/orbitofrontal cortices and periaqueductal gray matter ^9^ . Activity in these structures is delayed relative to actual cortical neurostimulation and becomes maximal during the hours that follow MCS arrest. Current hypotheses suggest that MCS may act through at least two mechanisms: activation of perigenual cingulate and orbitofrontal areas may modulate the emotional appraisal of pain, rather than its intensity, while top-down activation of brainstem periaqueductal gray matter may lead to descending inhibition toward the spinal cord. Recent evidence also points to a possible secretion of endogenous opioids triggered by chronic MCS ^9^ . In a recent article by Gordon et al, it was found with advanced precision functional mapping that the classic homunculus in the motor cortex is interrupted by regions with distinct connectivity, structure and function, alternating with effector-specific areas. These inter-effector regions exhibit strong functional connectivity to each other, as well as to the cingulo-opercular network and middle insula, critical for pain pathways. ^10^ This better knowledge of the networks that process pain will serve as the basis for the development in the future of more advanced and precise treatments for pain that does not respond to conventional treatments

## Conclusion

In correctly selected cases, where other treatments such as radiosurgery, thalamotomies or trigeminal nucleotomies, MVD, or percutaneous treatments are unfeasible, MCS needs to be considered. The patient obtained pain improvement in 50%, sustainably improving his quality of life.

## References

[1] Rasche D, Tronnier VM (2016). Clinical Significance of Invasive Motor Cortex Stimulation for Trigeminal Facial Neuropathic Pain Syndromes. Neurosurgery.

[2] Henssen DJHA, Witkam RL, Dao JCML, Comes DJ, Van Cappellen van Walsum AM, Kozicz T, van Dongen R, Vissers K, Bartels RHMA, de Jong G, Kurt E (2019). Systematic Review and Neural Network Analysis to Define Predictive Variables in Implantable Motor Cortex Stimulation to Treat Chronic Intractable Pain. J Pain.

[3] Lefaucheur JP, Hatem S, Nineb A, Ménard-Lefaucheur I, Wendling S, Keravel Y, Nguyen JP (2006). Somatotopic organization of the analgesic effects of motor cortex rTMS in neuropathic pain. Neurology.

[4] Burchiel KJ (2003). A new classification for facial pain. Neurosurgery.

[5] Callai EMM, Scarabelot VL, Fernandes Medeiros L, de Oliveira C, de Souza A, Macedo IC, Cioato SG, Finamor F, Caumo W, Quevedo ADS, Torres ILS (2019). Transcranial direct current stimulation (tDCS) and trigeminal pain: A preclinical study. Oral Dis.

[6] Hagenacker T, Bude V, Naegel S, Holle D, Katsarava Z, Diener HC, Obermann M (2014). Patient-conducted anodal transcranial direct current stimulation of the motor cortex alleviates pain in trigeminal neuralgia. J Headache Pain.

[7] Fontaine D, Hamani C, Lozano A (2009). Efficacy and safety of motor cortex stimulation for chronic neuropathic pain: critical review of the literature. J Neurosurg.

[8] Kurt E, Henssen DJHA, Steegers M, Staal M, Beese U, Maarrawi J, Pirotte B, Garcia-Larrea L, Rasche D, Vesper J, Holsheimer J, Duyvendak W, Herregodts P, van Dongen R, Moens M (2017). Motor Cortex Stimulation in Patients Suffering from Chronic Neuropathic Pain: Summary of Expert Meeting and Premeeting Questionnaire, Combined with Literature Review. World Neurosurg.

[9] Garcia-Larrea L, Peyron R (2007). Motor cortex stimulation for neuropathic pain: From phenomenology to mechanisms. Neuroimage.

[10] Gordon EM, Chauvin RJ, Van AN, Rajesh A, Nielsen A, Newbold DJ, Lynch CJ, Seider NA, Krimmel SR, Scheidter KM, Monk J, Miller RL, Metoki A, Montez DF, Zheng A, Elbau I, Madison T, Nishino T, Myers MJ, Kaplan S, Badke D'Andrea C, Demeter DV, Feigelis M, Ramirez JSB, Xu T, Barch DM, Smyser CD, Rogers CE, Zimmermann J, Botteron KN, Pruett JR, Willie JT, Brunner P, Shimony JS, Kay BP, Marek S, Norris SA, Gratton C, Sylvester CM, Power JD, Liston C, Greene DJ, Roland JL, Petersen SE, Raichle ME, Laumann TO, Fair DA, Dosenbach NUF (2023). A somato-cognitive action network alternates with effector regions in motor cortex. Nature.

